# A multilevel mixed effect analysis of neighbourhood and individual level determinants of risky sexual behaviour among young people in South Africa

**DOI:** 10.1186/s12978-022-01407-9

**Published:** 2022-05-12

**Authors:** Clifford Obby Odimegwu, Nebechukwu Henry Ugwu

**Affiliations:** 1 Demography and Population Studies Programme, Schools of Public Health and Social Sciences, University of the Witwatersrand, Johannesburg, South Africa; 2 Institute for Development Studies, University of Nigeria, Enugu Campus, Nigeria

**Keywords:** Neighbourhood and individual-level factors, Risky sexual behaviour, Youth, South Africa

## Abstract

**Background:**

Despite national and international commitments and efforts to prevent risky sexual behaviours, a high proportion of young people in South Africa are engaged in risky sexual behaviour. However, most efforts are currently directed toward addressing individual-level factors at the expense of not addressing neighbourhood-level determinants such as social disorganisation, contributing to risky sexual behaviour among young people in South Africa. This study investigated the multilevel factors of risky sexual behaviours among young people by gender in South Africa, using the lens of socio-ecological and social disorganisation frameworks.

**Methods:**

Data from a nationally representative sample of 1268 males and 2621 females aged 15–24 years, giving a total of, 3889 never-married youths, were drawn from the 2016 South Africa Demographic and Health Survey. Analysis was conducted using multilevel mixed-effect logistic regressions with random community-level effects.

**Results:**

Findings show that youth who were from a heterogeneous ethnic group (AOR = 0.49, CI: 0.35–0.67), household size of 5 + members (AOR = 0.78, CI: 0.54–1.15), community education (AOR = 0.97, CI: 0.72–1.32) were associated with low engagement in multiple sexual partnerships. Youths who were employed (AOR = 0.84, CI: 0.59–1.18), and from high-level community poverty (AOR = 0.76, CI: 0.58–1.00) were also associated with reduced odds of unprotected sex. In addition, older youth aged 20–24 years (AOR = 12.6, CI: 9.93–16.00); secondary education attainment (AOR = 1.01, CI 0.58–1.77); family structure (AOR = 1.37, CI: 0.75–1.15); Gauteng province (AOR = 1.45 CI: 0.92–2.28); residential mobility (AOR = 1.25, CI: 1.02–1.53), community media exposure to contraceptives (unprotected sex) (AOR = 1.38, CI: 1.09–1.76) were more likely to engage in risky sexual behaviour.

**Conclusion:**

The study revealed that neighbourhood and individual-level factors were important in explaining the factors associated with risky sexual behaviour among young people in South Africa. In addition, engagement in risky sexual behaviour was high, with minimal variation among young females and males in South Africa. It specifies that the practice of risky sexual behaviour is significantly associated with multilevel factors of social disorganisation that cut across gender. These results imply that there is a need to review policies of sexual risks reduction for each gender, which might help mitigate the adverse effects of social disorganisation for women and men youths in South Africa.

## Plain language summary

Risky sexual behaviour is most common among young people aged 15–24 years and is associated with an increase in sexually transmitted infections, including HIV/AIDS among this young productive age category. However, no adequate progress has been made in the reduction of these infectious diseases at the community level in South Africa. In this study, we investigate the effect of neighbourhood and individual-level factors leading to two measures of risky sexual behaviour; unprotected sex (non-condom use) and multiple sexual partnerships among young people. In this study, the target for young people in the South African context was because the country has a youthful population. Understanding the influence of neighbourhood and individual-level factors on the sexual behaviour of young people can help them live healthy as they transition to adulthood. We utilized cross-sectional data from the most recent South Africa Demographic and Health Survey, which was analyzed separately for males and females respectively, due to the cultural differences in sexual practices observed in many communities in South Africa. Our results confirmed an association between the neighbourhood and individual-level factors with youth engagement in risky sexual behaviour in South Africa. These results, imply that there is a need to review policies regarding the community-based interventions for sexual and reproductive health in other to reduce risky sexual behaviour among young people in South Africa.

## Background

**Fig. 1:**
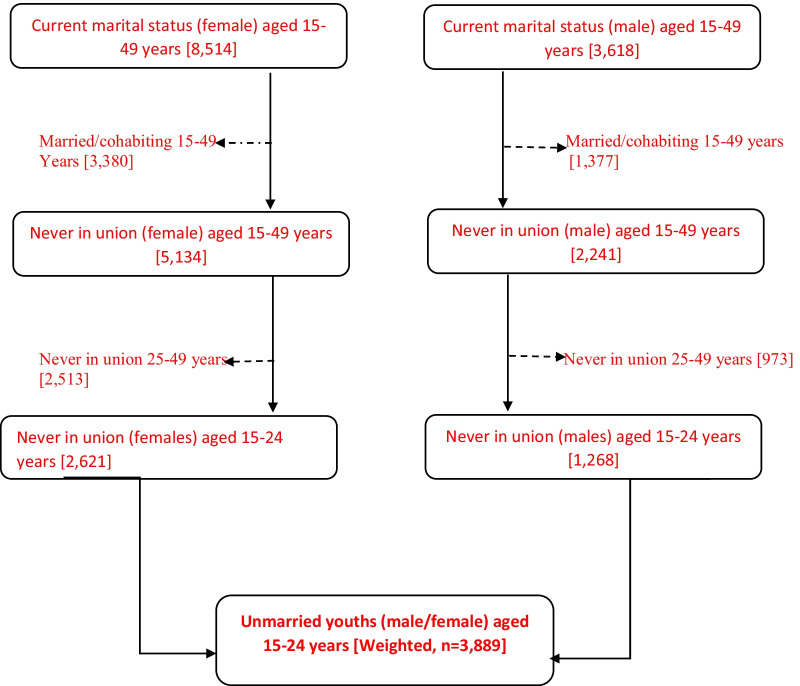
Procedure for sample selection by gender

Young people may appear to be in good health when compared to other age groups, they face unique health risks that may be detrimental not only to their immediate future but for the rest of their lives. Globally, it was estimated that only 40% of youth use any form of contraception, whereas 41.1% have had multiple sexual partners [1–3]. Despite the social, economic, demographic and health benefits of safe sex and adherence to consistent condom use, which is vital in promoting young people’s health and wellbeing. Estimates show that approximately 14 million young people die each year from Sexual and Reproductive Health (SRH) challenges [4, 5]. Sub-Saharan Africa (SSA) accounts for roughly 40% of the total [6, 7]. Furthermore, approximately 19 million new Sexually Transmitted Infections (STIs) cases are reported each year, with half of these cases occurring in youth aged 15–24 years [8]. It shows that the cause of this critical health condition in young people is their involvement in risky sexual behaviours [9, 10]. These behaviours include unprotected sex (non-condom use), inter-generational sex, early sexual debut, having multiple sexual partners and transactional sex. It has further resulted in unintended and early pregnancies for the females, increased sexually transmitted infections (STIs), including HIV/AIDS, among the youth (Fig. 1).

Risky sexual behaviour (unprotected sex and multiple sexual partnerships) by the youth establish to be higher in developing countries, especially in SSA, as compared to the developed countries [11–13]. As a result of anticipated consequences of risky sexual practices, several governments have resorted to creating awareness of risky sexual behaviour among the youth, with a view of delaying teenage pregnancies, reducing STIs, including HIV/AIDS, morbidity and mortality, and creating employment opportunities and education achievements among the youth. Some studies on factors associated with youth’s exposure to risky sexual behaviours in countries like Nigeria, Ghana, Kenya, Uganda, Rwanda and the Central African Republic [14–16]. The majority of these studies investigated the influence of individual-level factors on adolescents’ SRH [17–19]. Community poverty [20], community education [21], community media exposure to contraceptives [22], residential mobility [23, 24], province/region of residence [25], rural/urban resident [26], family structure [27], and ethnic diversity [28] are some of the notable factors associated with young people engagement in risky sexual practices.

Compared to some other countries bordering South Africa, like Botswana, Namibia and Mozambique, where statistics show that risky sexual behaviour among the youth was 17%, 23% and 25%, respectively, the 2020 high risks sexual report for South Africa was 27.8% [29–32]. It has led to high exposure to risky sexual behaviour and associated consequences [22, 33]. Despite government national programs and policies to address youths’ sexual health, such as the National Youth Policy 2015–2019, the Adolescent and Youth Policy 2012 and the National Strategic Plan for HIV/STIs and TB 2012–2016, to address the health needs of young people, and improve condom availability at the national level, youth in South Africa are more likely to engage in risky sexual behaviour compared to their adult counterparts [34–36].

The 2016 South Africa Demographic and Health survey (SADHS) reported contraceptive use among youth was lower than the married counterparts at 60% and 64.7%, respectively [37, 38]. Additionally, 27% of unmarried young people were having sex without any protection [37], and 56% of females and 61% of males reported having two or more sexual partners, which remains high to address the high rate of teenage pregnancies and HIV infections in South Africa. Risky sexual behaviour among youth has increased from 10 to 25% in 1998 to 45% in 2016 [39, 40]. However, HIV infections among the general population rise from 3.8 million in 2003 to 8.2 million in 2021 [41], while unwanted pregnancies among the youth rise from 30.2% in 2003 to 60% in 2021 [41, 42], but only 27.1% used any form of protection during sexual intercourse [37].

The study adopted socio-ecological and social disorganisation models to investigate the role and responsibilities of individual-level and neighbourhood-level risky sexual behaviours on young people. Individuals are viewed as nested within broader societal structures in the models, which describe the interactive characteristics of an individual and surroundings that underpin health outcomes [43, 44]. Ecological health research models are multifaceted, focusing on environmental, behavioural, and community policy decisions that assist individuals in making better choices in their daily lives [45]. They are unique in the study of overall mortality by taking the surroundings and their correlation to people into account at the intrapersonal, interpersonal, organizational, neighbourhood, and government policy levels. Socio-ecological models have shown impressive outcomes in attempting to prevent a wide range of public health issues [43, 46]. The studies observed, under socio-ecological models, that the social, physical, and cultural components of an environment have an accumulated influence on health [43, 47]. He upheld also that context is multifaceted because institutions and neighbourhoods are incorporated into the broader socio-economic structure, and also that the environmental context may significantly influence people’s health in different ways depending on their unique traditions and behaviours. Again, the social disorganization framework has argued that neighbourhoods with social disorganisation defects provide young people with fewer educational opportunities and a lack of employment opportunities to learn new skills or interact with positive adult role models [48, 49]. Social disorganisation is defined as the inability of community members to achieve shared values or to solve jointly experienced problems [50]. Prior research in South Africa has established a link between neighbourhood-level social disorganization and adolescent high-risk sexual behaviour Multiple Sexual Partnerships [(MSP) and unprotected sex] [51–53]. These studies do not investigate whether high-risk sex among youth is associated with the neighbourhood and individual differences within the communities. As a result, achieving long-term health improvement is most effective when all these factors (individual-level and neighbourhood-level factors) are addressed at the same time.

In this study, adopting a multilevel approach allows for an independent assessment of neighbourhood and individual-level factors that may contribute to young people’s continued exposure to high-risk sexual activities in South Africa. Using the neighbourhood theory of social disorganization. This study hypothesized that social disorganization as well as socio-ecological factors such as neighbourhood poverty, residential mobility, ethnic diversity, community media exposure to contraception. In addition, a family structure such as female-headed households, age, education attainments, and employment status act as risk factors for young people's exposure to risky sexual behaviour. Therefore, we also hypothesized, that the outcome would differ by region and place of residence, such as urban/rural settings due to differences in cultural values and norms surrounding sexuality among young people.

## Methods

The data for this study came from the 2016 South Africa Demographic Survey (SADHS). The 2016 SADHS was a nationally representative survey with a two-stage stratified sampling design. The SADHS 2016 is a cross-sectional study conducted by Statistics South Africa (Stats SA) in collaboration with the South African Medical Research Council (SAMRC) at the request of the National Department of Health (NDoH). The survey used the sampling frame from the Statistics South Africa Master Sample Frame (MSF), which was created using census 2011 enumeration areas (EAs). The census has a geographical hierarchy structure that links enumeration areas to administrative boundaries, so information is available in the survey at the municipal, district, and provincial levels. The survey provides the National Department of Health and policymakers with up-to-date demographic and health indicators from males and females. The full report includes a thorough explanation of the survey’s research design and methodology [36]. The analyses for this study included a weighted sample of 3889 young people (females—2621 and males—1268) who reported not being married at the time of the survey. The study relied on data from ‘young people,’ defined as those aged 15–24, as defined by the World Health Organization (WHO), and the term is used interchangeably with ‘adolescents’ and ‘youth’ [54]. Only the unmarried youths who reported to have engaged in risky sexual behaviours in the survey were included in the analysis.

### Variables and their measurements

*Dependent variables* Risky sexual behaviours expose youths to unwanted pregnancy or sexually transmitted infections. In this study, we considered two dependent variables of risky behaviours: (a) multiple sexual partners and (b) unprotected sex. Youths were considered to have engaged in risky sexual behaviours if they had multiple sexual partners in the 12 months preceding the survey and (b) unprotected sex without a condom in their last sexual intercourse. These variables were constructed using two questions. First, youths were asked, “how many sexual partners, excluding spouse, do you have in the 12 months preceding the survey?” Multiple sexual partners variable was coded “1” if the response to this question was one or more partners, and if otherwise, “0”. Second, participants were asked, "Did you use a condom during last sex with most recent partner?” Unprotected sex variable was coded “1” if youths reported not to use condom, and if otherwise, “0”. The 12-month reference period was useful for capturing the most recent behaviours and minimizing recall errors. The interest on number of sexual partners was because multiple sexual partnerships constitutes the key pathways through which young people can contract STIs and HIV infections which spreading sporadically across South African provinces.

*Independent variables* The independent variables were categorize into two: individual and neighbourhood level factors.

- Individual-level factors. These factors included age, employment status, educational attainment and household size.
  Individual factors which included youth’s age. For instance, risky sexual behaviour have been documented to be more common among younger persons than the older persons in the general population [51], therefore, age was categorized as 15–17, 18–19 and 20–24 years. Work status was assessed using a dichotomous variable coded as ‘no’ or ‘yes.’ The relationship between work status and risky behaviour can be difficult to predict at times because being employed can increase the proclivity to engage in risky behaviour by increasing exposure to opportunities that can be used to address occasional emotional and/or economic needs. It has been demonstrated that educational attainment is related to young people's sexual behaviours. Educated youth are more likely to be aware of reproductive health services, which may lead to healthier behaviours. The educational level was classified as ‘primary or less education, secondary education,’ or ‘higher education.’ Household size categorized as 1–4, 5–6 and 7 + members.
- Neighbourhood factors.
  These variables included: place of residence of was categorized as “urban and rural”. This was included because studies in South Africa have shown that youth in urban areas show higher levels for engaging in positive sexual behaviour due to the high level of campaigns around HIV/AIDS through the media, access to condom, including the availability of public washrooms, and easy access to sexual and reproductive health services, including ART. Province of residence (Western Cape, Eastern Cape, Northern Cape, Free State, Kwazulu-natal, North West, Gauteng, Mpumalanga and Limpopo). Community poverty: This was calculated using the percentage of households in the wealth index’s poorest quintile. Previous research on the effect of wealth status on youth sexual behaviour has yielded conflicting results. Wealth can sometimes act as an enabler or compensatory factor, and the effects vary by gender. Community poverty was classified as ‘low’ or ‘high.’ Ethnic diversity was expressed as a mixture of physical, behavioural, and cultural attributes. Also, it captures both the number of different groups in an area and the relative representation of each group [55]. It was categorized as “homogenous and Heterogeneous”. Residential mobility/instability: this was measured by percentage of residents in a community who have moved from current place of residence five years prior to the 2016 SADHS [56, 57]. It was categorized as “No and Yes”. The head of the household was used as a proxy for family structure and was classified as ‘male headed’ or ‘female headed.’ Community literacy level: the level at which a community can read and write. This was categorized as “low and high”. Contraceptive media exposure was measured by whether respondents reported hearing contraceptives messages on the radio or TV or reading them in magazines in the previous few months. A dichotomous variable was created and coded ‘1’ if the respondent answered ‘yes’ to at least two of the media forms and ‘0’ if they did not.

### Data analysis

In this study, the dataset were carefully checked for missing values before the analyses which were excluded and weighted with the appropriate sampling weights as per the Demographic and Health Survey (DHS) sampling scheme using Stata software (version 14). Data analysis was done at three stages. At the first stage of the analysis, we generated a descriptive survey of the variables using frequency distributions for categorical variables. At the second stage, we used a bivariate multilevel logistic regression to investigate the association between individual and neighbourhood-level factors with risky sexual behaviour and a p-value < 0.05 set as a significant level at 95% confidence level. At the third stage, a multivariate two-level mixed-effects logistics regression model was applied to investigate the effects of neighbourhood determinants on the two outcome variables with youth at level 1 being nested within neighbourhoods at level 2. The analysis at the third stage took four models; model 1 (empty model) was fitted without any of the explanatory variables to test the random variability in the intercept and show the total variance in exposure to risky sexual behaviour (multiple sexual partners and non-condom use) among the youth in different neighbourhoods. Model 2, investigated the effects of individual-level determinants. Model 3 investigated the effects of neighbourhoods and model 4 investigated the effects of both individual and neighbourhood-level determinants interchangeably with the results of fixed effects being shown as odds ratios at 95% confidence level. The inter-cluster correlation coefficient (ICC) for each model was calculated to explain the proportion of variation and to compare the successive models. These values were obtained from ICC = σ^2^/ (ðσ^2^ + þπ^2^ /3), where σ^2^ is the estimated community level variance while π^2^ /3 is the household variance. The Proportional Change in Variance (PCV) was also computed for each model with respect to the empty model to show the power of the factors in the models in explaining the outcome variable. The PCV was obtained from PCV = (V − Vi)/ Ve, where Ve is variance of risky sexual behaviour in the empty model and Vi is variance in successive models. The two-level multilevel model with a binary response variable for a youth i living in neighbourhood j, is represented as:

$$\mathrm{Log }[\mathrm{\pi ij}/1-\mathrm{\pi ij}] =\upbeta 0 +\upbeta 1\mathrm{X}1\mathrm{ij }+ \dots +\mathrm{ \beta nXnij }+\upupsilon 0\mathrm{ j }+\mathrm{ \varepsilon ij}$$

where: πij is the probability that the ith never-married youth in the jth neighbourhood was engaged in risky sexual behaviour (1 − πij) is the probability that he/she was not involved in risky sexual behaviour, β0 is the log odds of the intercept: β1, β2^+^, βπ are the effect sizes by the individual and neighbourhood-level variables: X1ij, X2ij, X1ij are the independent variables at individual and neighbourhood level: υ0 j and εij are random errors at neighbourhood and individual levels respectively.

## Results

### Distribution of individual-level characteristics

Table 1 presents the selected individual characteristic of never-married youth 15–24 years who were nested within 750 communities with number of the youth ranges from 4 to 20 (average of 10). The results indicate that 44.3% females and 44.5% males aged 20–24 years were reported to have engaged in risky sexual behaviour within the 5 years prior the survey. The table further shows that 88% females and 82% males of the youth with a secondary education were exposed to risky sexual behaviour, 91.4% female and 83.7% male youth were not employed. Additionally, 37.3% and 45.7% of the female and male youth are from a household size of 1 to 4 members.

**Table 1:** Distribution of youth by background characteristics

	Female (N = 2621)		Male (N = 1268)	
	Freq	Percent	Freq	Percent
Respondent’s age				
15–17	904	34.49	419	33.04
18–19	557	21.25	285	22.48
20–24	1160	44.26	564	44.48
Education attainments				
Primary and less education	157	5.99	172	13.56
Secondary	2306	87.98	1,045	82.41
Higher	158	6.03	51	4.02
Employment status				
No	2396	91.42	1061	83.68
Yes	225	8.58	207	16.32
Household size				
1–4	977	37.28	579	45.66
5–6	750	28.62	310	24.45
7 +	894	34.11	379	29.89

Source: SADHS 2016

### Distribution of neighbourhood-level characteristics

Table 2, indicates the distribution of the youth by selected community characteristics. The table shows that 89.3% females and 91.1% males were from a homogenous ethnic group, while half of the females’ respondents and 47.5% males’ youth were from a household headed by a female. The results in the table also show that half (53.1%) females and 46.8% male youth lived in rural areas, while Kwazulu-natal province had the highest youth reported to have engaged in risky sexual behaviour at 19.4% females and 17.3% males’ respectively. The results further shows that 20% females and 21.4% males highlighted that they changed residence while more than half of both youth who lived in a community that had a high proportion of a high media exposure to contraceptive (57.2% females and 61.2% males), high poverty (45% females and 47.6% males), and a high community education (7.6% females and 16.3% males) respectively.

**Table 2:** Distribution of respondent by community characteristics

	Female (N = 2621)		Male (N = 1268)	
Ethnic/Racial diversity				
Homogenous	2340	89.28	1154	91.01
Heterogeneous	281	10.72	114	8.99
Family structure				
Male	932	35.56	602	47.48
Female	1689	64.44	666	52.52
Place of residence				
Urban	1393	53.15	594	46.85
Rural	1228	46.85	674	53.15
Province of residence				
Western cape	175	6.68	61	4.81
Eastern cape	349	13.32	191	15.06
Northern cape	206	7.86	83	6.55
Free state	260	9.92	135	10.65
Kwazulu-natal	509	19.42	220	17.35
North west	218	8.32	123	9.7
Gauteng	206	7.86	99	7.81
Mpumalanga	331	12.63	154	12.15
Limpopo	367	14	202	15.93
Residential mobility				
No	2071	79.02	997	78.63
Yes	550	20.98	271	21.37
Community poverty				
Low	1441	54.98	665	52.44
High	1180	45.02	603	47.56
Community media exposure to cp				
Low	1124	42.88	493	38.88
High	1497	57.12	775	61.12
Community education				
Low	199	7.59	207	16.32
High	2422	92.41	1061	83.68

Source: SADHS 2016

### Bivariate analysis of risky sexual behaviour

The unadjusted odds ratio results in Table 3 indicate that never-married youth aged 18–19 or 20–24 years, females working, those with a secondary or higher education attainments, coming from a female headed household, those lived in rural areas, province of residence had increased odds of multiple sexual partnerships. In addition, compared to those from a homogenous ethnic group, or those who have not changed residence, or those without media exposure to contraceptives were more likely to have multiple sexual partners for both male and female youth. Table 3, also indicate that youth aged 20–24 years, those with a secondary or higher education, and those living in a household with 5–6 or 7 + members were significantly likely not to use condom at their last sexual activities for across gender.

**Table 3:** Association of risky sexual behaviour with selected characteristics

Individual characteristics	Female		Male	
	MSPs	Unprotected sex	MSPs	Unprotected sex
	OR (95%CI)	OR (95%CI)	OR (95%CI)	OR (95%CI)
Age				
15–17Ref	1	1	1	1
18–19	4.34 (3.46–5.44)***	1.27 (0.89–1.78)	4.05 (2.94–5.67)***	0.86 (0.48–1.51)
20–24	11.64 (9.46–14.32)***	1.12 (0.83–1.50)*	12.58 (9.24–17.13)***	0.70 (0.43–1.15)*
Education attainment				
Primary or less edu	1	1	1	1
Sec	1.79 (1.29–2.49)***	1.98 (1.20–3.26)**	2.86 (2.05–3.98)***	3.40 (2.03–5.72)***
Higher	3.90 (2.43–6.28)***	2.62 (1.40–4.88)**	25.69 (7.69–85.95)***	2.38 (1.07–5.30)**
Employment status				
No	1	1	1	1
Yes	3.06 (2.21–4.25)***	0.92 (0.66–1.26)	3.76 (2.55–5.54)***	1.25 (0.82–1.89)
House size				
1–4	1	1	1	1
5–6	0.89 (0.74–1.08)	0.83 (0.64–1.09)*	0.67 (0.50–0.89)**	1.65 (1.06–2.57)**
7 +	1.08 (0.89–1.29)*	0.67 (0.52–0.86)***	0.76 (0.58–0.99)**	1.63 (1.09–2.44)**
Family structure				
Male	1	1	1	1
Female	1.47 (1.25–1.73)***	0.91 (0.73–1.44)*	0.73 (0.58–0.92)**	1.16 (0.83–1.62)**
Place of residence				
Urban	1	1	1	1
Rural	1.10 (0.94–1.29)**	1.56 (1.27–1.92)***	0.90 (0.72–1.14)***	1.22 (0.87–1.70)**
Province of residence				
Western cape	1	1	1	1
Eastern cape	2.76 (1.89–4.00)***	1.02 (0.60–1.70)	2,22 (1.23–4.10)***	1.78 (0.72–4.16)**
Free state	1.23 (0.82–1.84)*	0.72 (0.39–1.33)	1.39 (0.72–2.71)**	0.74 (0.25–2.18)**
Northern cape	1.36 (0.93–2.00)*	0.53 (0.29–0.95)**	1.40 (0.77–2.59)**	0.77 (0.29–2.03)
Kwazulu-natal	1.36 (0.96–1.92)	1.12 (0.67–1.87)	1.23 (0.69–2.16)**	0.93 (0.37–2,29)*
North West	1.99 (1.83–2.99)***	0.72 (0.40–1.28)	1.87 (0.99–3.49)**	0.36 (0.12–1.04)**
Gauteng	2.23 (1.48–3.36)***	0.44 (0.24–0.79)**	2.34 (1.20–4.53)**	0.78 (0.29–2.10)*
Mpumalanga	2.58 (1.77–3.76)***	0.75 (0.44–1.27)	1.65 (0.90–2.99)***	0.68 (0.25–1.69)***
Limpopo	1.27 (0.88–1.82)	0.81 (0.47–1.40)	1.39 (0.70–2.46)***	0.76 (0.30–1.91)***
Ethnic diversity				
Homogenous	1	1	1	1
Heterogeneous	0.43 (0.34–0.56)***	1.44 (0.97–2.13)*	0.43 (0.29–0.64)***	1.35 (0.70–2.50)**
Residential mobility				
No	1	1	1	1
Yes	1.32 (1.09–1.60)**	0.77 (0.60–0.99)**	1,95 (1.45–2.63)***	1.10 (0.76–1.61)**
Community poverty				
Low	1	1	1	1
High	1.26 (1.08–1.47)***	1.60 (1.30–1.98)***	1.14 (0.90–1.43)***	1.59 (1.14–2.22)***
Community media exposure to CP				
Low	1	1	1	1
High	0.89 (0.76–1.04)*	0.64 (0.52–0.79)***	1.45 (1.15–1.82)***	0.50 (0.36–0.71)***
Community education				
Low	1	1	1	1
High	0.95 (0.70–1.27)*	0.74 (0.50–1.09)*	1.60 (1.19–2.16)***	0.55 (0.35–0.86)**

Ref., reference category. *p < 0.1; **p < 0.05; ***p < 0.01

Further, in regards to community factors, family structure (female headed home) in which male youth lived, province of residence, ethnic diversity, residential mobility had increased odds of non-condom use among the male youth, while ethnic diversity, residential mobility, community poverty, media exposure to contraceptives were highly associated with non-condom use among the female youth.

### Individual and neighbourhood level characteristics associated with multiple sexual partnerships among the youth

Table 4 shows the results of eight models, four models for each gender, which is female and male youths in the exposure to have multiple sexual partners. Model 1 had only the dependent variable with the results showing a statistically significant variability in the odds of MSP between communities (τ = 0.41, p-value < 0.001) and (τ = 0.74, p-value < 0.001) for the females and males respectively. The ICC in this model indicated that 48% and 14% of the total variance in having multiple sexual partners among the female and male youths was attributed between communities. In model 2, individual-level variables were included. The results showed that the age of the youths, employment status, and educational attainment was significantly associated with multiple sexual partners across gender. Meanwhile, only household size was not associated with male involvement in MSP. The ICC in this model indicated that females 6% and males 21% of the variation in multiple sexual partners was attributed to differences across neighbourhoods whereas a PCV implied that females 24% and males 47% of the variance in having multiple sexual partners across neighbourhood was explained by these individual characteristics.

**Table 4:** Multilevel mixed effects analysis of individual and neighbourhood factors associated with MSP among young male and female

Variables	Female			Male		
	Model 1	Model 2	Model 3 (full model)	Model 1	Model 2	Model 3 (full model)
	AOR (95% CI)	AOR (95% CI)	AOR (95% CI)	AOR (95% CI)	AOR (95% CI)	AOR (95% CI)
Age						
15–17RC	1		1	1		1
18–19	4.56 (3.58–5.82)***		4.54 (3.58)***	4.86 (3.23–7.29)***		4.71 (3.16–7.03)***
20–24	12.62 (9.93–1602)***		12.08 (9.60–15.19)***	14.01 (9.09–21-59)***		13.42 (8.70–20.68)***
Education						
Primary or less education. RC			1	1		1
Secondary	1.21 (0.81–1.80)		1.42 (0.93–2.14)*	2.09 (1.34–3.28)***		2.02 (1.25–3.26)***
Higher	1.01 (0.58–1.77)		1.36 (0.76–2.41)	7.12 (1.88–21.03)**		6.50 (1.66–25.56)
Employment status						
No RC	1		1	1		1
Yes	1.28 (0.89–1.85)		1.44 (0.99–2.08)*	1.56 (0.95–2.54)		2.05 (1.24–3.41)**
Household number						
1–4	1		1	1		1
4–5	3.05 (0.83–1.32)		1.07 (0.85–1.34)	0.79 (0.54–1.15)		0.83 (0.57–1.21)
7 +	1.20 (0.97–1.50)*		1.16 (0.93–1.46)*	0.80 (0.55–1.15)		0.85 (0.59–1.22)
Family structure						
Male headed		1	1		1	1
Female headed		1.37 (1.16–1.62)***	1.13 (0.93–1.38)		0.73 (0.55–0.95)**	0.90 (0.66–1.23)
Place of residence						
Urban		1	1		1	1
Rural		0.93 (0.75–1.15)	0.98 (0.77–1.26)		0.80 (0.56–1.15)	0.99 (0.64–1.53)
Province of residence						
Western Cape		1	1		1	1
Eastern Cape		1.78 (1.65–2.71)**	1.88 (1.16–3.06)**		1.57 (0.71–3.46)	3.27 (1.28–8.33)*
Northern cape		1.06 (0.69–1.61)	1.05 (0.65–1.70)		1.20 (0.53–2.72)	1.66 (0.64–4.32)
Free State		0.88 (0.57–1.35)	0.77 (0.47–1.26)		0.90 (0.40–2.03)	1.57 (0.60–4.06)
Kwazulu-natal		0.84 (0.56–1.26)	0.67 (0.41–1.06)		0.76 (0.35–1.65)	0.85 (0.34–2.08)
Northern west		1.32 (0.84–2.07)	1.32 (0.78–2.22)		0.94 (0.41–2.16)	1.26 (0.47–3.37)
Guanteng		1.45 (0.92–2.28)*	1.33 (0.79–2.23)		1.16 (0.49–2.72)	1.66 (0.60–4.55)
Mpumalanga		1.63 (1.05–2.54)*	1/71 (1.03–2.84)*		0.95 (0.43–2.12)	1.40 (0.55–3.63)
Limpopo		0.79 (0.51–1.24)	0.71 (0.43–1.17)		0.79 (0.35–1.75)	0.94 (0.37–2.38)
Ethnic diversity						
Homogenous		1	1		1	1
Heterogenous		0.49 (0.35–0.67)***	0.42 (0.29–0.61)***		0.33 (0.19–0.59)***	0.24 (0.12–0.46)***
Community poverty						
Low		1	1		1	1
High		1.11 (0.90–1.36)	1.21 (0.95–1.53)		1.28 (0.93–1.77)	1.18 (0.80–1.73)
Community media exposure to contraception						
Low		1	1		1	1
High		1.00 (0.44–1.20)	0.90 (0.74–1.10)		1.74 (1.31–2.30)***	1.41 (1.01–1.97)**
Community education						
Low		1	1		1	1
High		0.97 (0.72–1.32)	0.86 (0.59–1.24)		1.43 (1.01–2.04)*	1.30 (0.84–2.02)
Residential mobility						
No		1	1		1	1
Yes		1.25 (1.02–1.53)**	1.09 (0.86–1.38)		1.79 (1.25–2.54)***	1.30 (0.86–1.73)
Random effects						
Community variance (SE)	0.46 (0.02)***	0.48 (0.66)***	0.50 (1.06)***	094 (0.16)***	0.61 (0.14)***	0.74 (0.17)***
VPC = ICC (%)	0.060608	0.080608	0.10807	0.21295	0.10176	0.141975
Explained variation PCV (%)	− 24.57	86	100	− 47.7182	29.41192	1.515404
Model fit statistics						
Log Likelihood	− 1467.84	− 1736.63	− 1410.81	− 667.542	− 799.985	− 636.892
AIC	2953.682	3507.252	2869.619	1353.083	1633.97	1321.783

Ref., reference category. *p < 0.1; **p < 0.05; ***p < 0.01

In model 3, only neighbourhood level determinants were added and results revealed that female youth residing in a female-headed home, province of residence, ethnic diversity and community media exposure to contraceptives was positively associated with having multiple sexual partners. In addition, community poverty, residential mobility and residing in rural areas were also positively associated with multiple sexual partners. Province of residence, such as residing in Eastern Cape, Northern Cape and Gauteng were positively associated with having MSP among the male youths. Surprisingly, male youth residing in a female-headed household, those living in rural areas and ethnic diversity were not associated with having multiple sexual. Additionally, residential mobility, media exposure to contraceptives, community poverty, and community education was positively associated with having multiple sexual partners among the males. The ICC in this model showed that the differences between neighbourhoods accounted for 8% and 10% of the variation in having multiple sexual partners among the female and male youth, while PCV indicated that 86% and 29% of the neighbourhood variation in multiple sexual partnerships was explained by neighbourhood level characteristics for both female and male youth respectively.

Model 4, included both the individual and neighbourhood level determinants. In this model, the variation in the odds of exposure to multiple sexual partnerships remained statistically significant for females (τ = 0.50, p-value < 0.001) and males (τ = 0.74, p-value < 0.001) with an estimated 10% and 14% variability in having multiple sexual partners among female and male youth, was attributed to differences between neighbourhoods. Additionally, 100% and 51% of the variations in multiple sexual partners across neighbourhoods are explained by both individual and neighbourhood-level determinants among the female and males respectively. The results in model 4 show that an increase in youth’s age increases the odds of having multiple sexual partners. Specifically, female youth aged 20–24 years (AOR = 12.08, CI = 9.60–15.19) and male youth aged 20–24 years (AOR = 13.42, CI = 8.70–20.68) were more likely to have multiple sexual partners compared to those less than 20 years. The result also indicates that female youth (AOR = 1.44, CI = 0.99–2.08) and male youth (AOR = 2.05, CI = 1.24–3.41) with paid employment were more likely to have multiple sexual partners compared to those not working. In terms of education, female youth with higher education attainment (AOR = 1.36, CI = 0.76–2.41) and male youth with higher education attainment (AOR = 6.50, CI = 1.66–25.56) had increased odds of exposure to having multiple sexual partners. Additionally, for the neighbourhood factors, female youth who reported residing in a community with a high poverty level likely (AOR = 1.21, CI = 0.95–1.53), and male youths (AOR = 1.18, CI = 0.80–1.73) were more to multiple sexual partners compared to those reported to come from communities with low poverty levels. The results in Table 4, further indicate that female youth who have changed residences (AOR = 1.09, CI = 0.86–1.38) and male youth (AOR = 1.03, CI = 0.86–1.73) were more likely to engage in multiple sexual partnerships, while only community media exposure to contraceptives (AOR = 0.40, CI = 0.74–1.10) were associated with lower odds of multiple sexual partners among the female youth.

### Individual and neighbourhood level characteristics associated with unprotected sex among the youth

Table 5, shows the results of eight models, four models each in unprotected sex (non-condom use) for both female and male youth. Model 1 had only the outcome variable with the results showing a statistically significant variability in the odds of condom use at the last sex between communities, females (τ = 0.48, p-value < 0.001) and males (τ = 0.72, p-value < 0.001) respectively. The ICC in the model indicates that 6% and 8% of the total variance in not using condoms among the female and male youth was attributed between neighbourhoods. In model 2 for both genders, individual-level determinants were included. The results showed that age and education attainments increase the odds of unprotected sex for the female youth. For the male youth, employment status and household size increase the odds of non-condom use. While working and household size were not associated with unprotected sex among the females. Only age was not associated with unprotected sex among the male youth. The ICC in this model indicated that 5% and 10% of the variation in unprotected sex was attributed to differences across neighbourhoods whereas PCV implied that 24% and 36% of the variance in not using a condom during sexual activities across neighbourhoods was explained by these individual characteristics for the female and male youth respectively. In model 3, only neighbourhood determinants were added and the results revealed that female youth residing in a female-headed home, residential mobility, and community media exposure to contraceptives, community education and the province of residence were associated with unprotected sex among females and male youth. The ICC in this model showed that the differences between neighbourhoods accounted for 16% and 28% of the variation in non-condom use among the female youth, while PCV indicated that 76% and 79% of the neighbourhood variation in non-condom use was explained by neighbourhood level determinants for the female and male youth respectively.

**Table 5:** Multilevel mixed effects analysis of individual and neighbourhood factors associated with unprotected sex (condom use) among young male and female

Variables	Female			Male		
	Model 1	Model 2	Model 3 (full model)	Model 1	Model 2	Model 3 (full model)
	OR (95% CI)	OR (95% CI)	OR (95% CI)	OR (95% CI)	OR (95% CI)	OR (95% CI)
Age						
15–17RC	1		1	1		1
18–19	1.23 (0.86–1.77)		1.17 (0.81–1.69)	0.65 (0.35–1.22)		0.58 (0.31–1.09)
20–24	1.05 (0.76–1.44)		0.94 (0.68–1.29)	0.53 (0.29–0.93)**		0.46 (0.26–0.82)
Education						
Primary or less edu RC	1		1	1		1
Secondary	1.80 (1.06–3.06)*		1.55 (0.89–2.67)	3.93 (2.19–7.05)***		3.06 (1.62–5.77)***
Higher	2.32 (1.19–4.54)**		1.70 (0.85–3.42)	2.80 (1.16–6.78)**		2.12 (0.01–5.55)
Employment status						
No	1		1	1		1
Yes	0.84 (0.59–1.18)		0.87 (0.61–1.23)	1.64 (1.02–2.63)**		1.54 (0.96–2.47)
Household number						
1–4	1		1	1		1
4–5	0.83 (0.63–1.10)		0.86 (0.65–1.15)	1.69 (1.05–2.74)**		1.67 (1.04–2.68)*
7 +	0.68 (0.52–0.89)**		0.79 (0.59–1.03)	1.69 (1.09–2.63))**	1.69 (1.09–2.63)**	1.60 (1.03–2.48)**
Family Structure						
Male		1	1		1	1
Female		1.19 (0.93–1.50)*	1.15 (0.89–1.46)		1.19 (0.82–1.71)	1.09 (0.75–1.58)
Place of residence						
Urban		1	1		1	1
Rural		0.77 (0.58–1.03)	0.77 (0.58–1.03)		0.90 (0.57–1.45)	``0.96 (0.59–1.54)
Province of residence						
Western Cape		1	1		1	1
Eastern Cape		1.16 (0.63–2.11)	1.24 (0.68–2.26)		0.69 (0.25–1.91)	0.55 (0.24–1.36)
Northern cape		1.65 (0.87–3.13)	1.74 (0.92–3.32)		1.28 (0.41–4.02)	1.26 (0.56–3.04)
Free State		1.61 (0.84–3.10)	1.69 (0.88–3.27)		1.13 (0.39–3.32)	1.12 (0.38–2.04)
Kwazulu-natal		0.95 (0.52–1.73)	1.03 (0.56–1.87)		1.15 (0.40–3.22)	1.02 (0.38–2.07)
Northern west		1.46 (0.75–2.80)	1.56 (0.80–3.02)		2.73 (0.82–9.07)	2.67 (0.77–8.05)
Guanteng		1.99 (1.03–3.86)**	2.06 (1.06–3.98)*		1.16 (0.38–3.53)	1.14 (0.29–2.88)
Mpumalanga		1.54 (0.83–2.87)	1.63 (0.87–3.04)		1.53 (0.52–4.55)	1.48 (0.55–3.01)
Limpopo		1.56 (0.82–2.97)	1.62 (0.85–3.09)		1.44 (0.49–4.28)	1.41 (0.46–3.04)
Ethnic diversity						
Homogenous		1	1		1	1
Heterogenous		0.58 (0.36–0.96)*	0.59 (0.36–0.97)*		0.71 (0.32–1.57)	0.69 (0.47–1.03)
Community poverty						
Low		1	1		1	1
High		0.76 (0.58–1.00)*	0.79 (0.59–1.04)		0.78 (0.50–1.19)	0.88 (0.41–1.02)
Community media exposure to CP						
Low		1	1		1	1
High		1.38 (1.09–1.76)	1.37 (1.06–1.75)*		1.50 (1.04–2.19)**	1.31 (1.05–0.91)
Community education						
Low		1	1		1	1
High		1.17 (0.78–1.74)	1.05 (0.69–1.59)		1.55 (0.96–2.49)	1.33 (0.86–0.23)
Residential mobility						
No		1	1		1	1
Yes		1.29 (0.99–1.70)	1.22 (0.92–1.62)		0.995 (0.62–1.46)	0.10 (0.54–0.52)
Random effects						
Community variance (SE)	0.42 (0.13)***	0.23 (0.20)***	0.19 (0.25)***	0.56 (0.28)***	0.31 (0.42)**	0.47 (0.38)*
VPC = ICC (%)	0.051111	0.016046	0.011144	0.088115	0.028488	0.43883
Explained variation PCV (%)	24.58531	76.32492	83.55648	36.18729	79.36892	86.67462
Model fit statistics						
Log Likelihood	− 974.343	− 954.328	− 949.757	− 405.574	− 404.592	0.032714
AIC	1966.686	1942.656	1947.514	829.147	843.1848	844.245

Ref., reference category. *p < 0.1; **p < 0.05; ***p < 0.01

Model 4, included both the individual and neighbourhood-level determinants. In this model, the variation in the odds of non-condom use remained statistically significant, female (τ = 0.19, p-value < 0.001) and male (τ = 0.47, p-value < 0.001) with an estimated 11% and 43% variability in non-condom use attributed to differences between neighbourhoods with 86% of the variation in non-condom use across neighbourhoods being explained by both individual and neighbourhood-level determinants for both male and female youth. The results in model 4 show that an increase in age decreases exposure to unprotected sex for the female youth (AOR = 0.94, CI = 0.68–1.29) and male youth (AOR = 0.94, CI = 0.68–1.29) respectively. For instance, female youth aged 18–19 years were more likely (AOR = 1.17, CI = 0.81–1.69) male youth (AOR = 0.58, CI = 0.31–1.09) to unprotected sex compared to those more than 20 years. In terms of education attainment, results indicate that female youth with secondary education (AOR = 1.55, CI = 0.89–2.67) or higher education (AOR = 1.70, CI = 0.85–3.42) had increased odds of non-condom. Results further indicate that male youth with a secondary education (AOR = 3.06, CI = 2.19–7.05) or a higher education (AOR = 2.12, CI = 0.01–5.55) had increase odds of unprotected sex. In addition, the neighbourhood effects show that female youth who reported residing in a female-headed household (AOR = 1.15, CI = 0.89–1.46), community education (AOR = 1.05, CI = 0.69–1.59), those exposed to media on contraceptive use (AOR = 1.37, CI = 1.06–1.75), residential mobility (AOR = 1.22, CI = 0.92–1.62) were more likely not to use a condom while residing in Gauteng province were double likelihood (AOR = 2.06, CI = 1.06–1.59) of non-condom use among the female youth. For the male youth, the neighbourhood effects show that residing in a female-headed household (AOR = 1.09, CI = 0.75–1.58), community education (AOR = 1.33, CI = 0.86–0.23), those exposed by media to contraceptive use (AOR = 1.31, CI = 1.05–0.91), were more likely not to use a condom during sexual activities, while residing in North West province were double likelihood (AOR = 2.67, CI = 0.77–8.05) of non-condom use among the male youth.

## Discussion

In this study, we investigated neighbourhood and individual-level determinants of risky sexual behaviours among never-married youth aged 15–24 years in South Africa. Our results showed that both individual and neighbourhood level factors were significant in explaining the factors associated with exposure to risky sexual behaviour among youth in South Africa. Results further showed that the age of the youth (males and females), employment status, household size, ethnic diversity, community media exposure to contraceptives, family structure (female-headed home), residential mobility and province of residence had a significant association with risky sexual behaviours among the youth.

The results show that older youth are more likely to have multiple sexual partners compared to younger adolescents. Specifically, youth aged 18–24 years engaged in multiple sexual partners than those aged 15–17 years. The explanation for these age differentials could be attributed to the fact that older youth assumed to have had more knowledge of the most common infections associated with sexual risk practices. This was evident in a study done on young people in Zimbabwe [58], which reported that young adults over 20 years reported having engaged in risky sexual activities. The finding was attributed to having confidence in making decisions about their sexual lives. Unlike young people below 20 years that were afraid to report involvement in risky sexual activities, due to avoid being identified by a closed relatives within their community. Which most times, frowned at premarital sex or if they are still living in a household with family members who are unaware of their sexual activities. In South Africa, the consequences of under-reporting risky sexual behaviour among the younger youth aged 10 to 19 years were due to abuse and discrimination against teenage sex within the communities. Most especially if they are still living in a household with family members who are unaware of their sexual activities. Therefore, without complete and accurate information about the sexual risks involvement among young people, efforts to protect and prevent the spread of sexual infections will be severely challenged.

Our results also indicate that highly educated youth were more likely to engage in risky sexual behaviour than less educated ones. The possible explanation for these results could be that education empowers young people with information and increases bargaining power in decision making in a sexual relationship. However, in South Africa, low utilization of knowledge obtained in schools among this young population is not entirely unaccepted as a recent cross-sectional study of the population shows that 87.3% of the participants engaged in risky sexual activities [59]. Therefore, more studies are needed to investigate factors that may increase the risk for HIV among educated youth, especially, because they know and are aware of the consequences of HIV infections.

Meanwhile, reported multiple sexual partnerships was associated with an increased risk of HIV infections among the female youth. For instance, female youth residing in a household with five members and above increased the odds likelihood of multiple sexual partnerships by three times. These odds are high compared to a study conducted on female youth aged 15–24 years in Zambia, which showed odds likelihood of having multiple sexual partners by 1% from a household of 5 members and above [60]. This study reveals a negative association between lack of household supports and risky sexual practices. This lack of household support especially living in a household with more than five members and above was more evident among the female youth. Other studies have attributed that lack of food, materials and housing distress are risks factors for risky sexual involvements among females [61–64]. The consequence of these findings from extant literature is the opportunities offered by other environments (e.g., schools, clubs, etc.) to this young female population to engage in risky sexual behaviour. For instance, the school environment could provide a female student with freedom to “fly out of the cage” which could encourage her to have multiple sexual partners to “meet up” with her desired “lifestyles” on campus.

Moreover, our findings indicate that community poverty is a predominant factor in youth desires to engage in risky sexual behaviour. Male and female youth living in communities marked by high poverty were more likely to engage in risks sexual activities compared to those who lived in communities with low poverty, and this finding corroborated with other studies [20, 26–28, 65]. The possible explanation for these findings is that poor communities may offer opportunities for lack of access to healthcare, food and housing, school drop-out, unemployment due to lack of education and further engenders lack of supervision and monitoring of youth activities. These findings imply that youth living in a poor community may not have adequate knowledge and information to protect themselves from the consequences of risky sexual behaviour. Thus, without complete and accurate information about risks associated with youth sexual and reproductive health, government efforts to protect the youth and prevent the spread of HIV infections at the community level will be severely challenged.

In communities with residents having a history of residential mobility increased the odds of young people engaging in risky sexual activities. Our findings are in line with other studies, which shows that residential instability comes with “cutting of social connections” to friends and support networks, excessive change of schools with a diversity of cultural norms and values that encourage young people to engage in risky sexual behaviour [23, 24, 66]. Consequently, constant change of residence could heighten exposure to engage in risky sexual behaviour among the youth, thus increasing HIV infection risks among them. These findings suggest that much need to be done on the effects of fragmented education on youth sexual behaviour in South Africa.

Concerning community education, youth from a community with a higher proportion of inhabitants who can read and write were more likely to engage in risky sexual behaviour. This result is surprising because a community with a high proportion of literate parents may provide information on positive sexual behaviour among the youth. The possible explanation for this finding is that progress on the policies and programs of adolescent sexual health in South Africa, may not be monitored at the community levels. Imply a need to set up a committee that will educate the youth on the risk of sexual practices, especially at the community square where they usually congregate. Our study partly corroborated with other studies [21, 22].

Our findings show that youth who indicated that they reside in a female-headed home were more likely to engage in risky sexual behaviour than those from the male-headed household. In South Africa, a recent publication shows that 41.8% of households were female-headed, with Eastern Cape Province having the highest proportion with over 50% female-headed households residing in the rural area [41]. This finding may be due to an increase in divorce and single-parent families. Although the divorce rate in South Africa is low at 17.6%, however, almost one in five marriages end up in divorce [41]. Although awareness about sexual risks reduction is high in South Africa, the knowledge is not well utilized due to limited monitoring and control of youth activities at the community level. These findings have great implications for STIs prevention among the youth, especially females, who might not be closely monitored by their mothers, unlike their male counterparts. This is because there is a tendency for young females to meet with their sexual partners outside their homes than the male youth, who most often invite their sexual partners outside to the family. Similar studies have documented how coming from a disrupted family encourage young people to engage in risky sexual activities [27, 67].

Although the association between individual access to reproductive health and condom availability by young people is high, we found that community media exposure to contraceptive use is associated with risky sexual behaviour. Youth in communities with a high media exposure to contraceptive messages (through Radio, TV, Newspapers etc.) had an increased odds of exposure to risky sexual behaviour by 1% across gender. Though, the percentage is low compared to a study conducted in Nigeria which showed about 3% of youth with exposure to mass media engaged in risky sexual activities [68]. This finding suggests that mass media campaigns and community gathering activities can help to increase awareness of risky sexual behaviour among youth. This finding is corroboration with other studies [22, 68] on media exposure to risky sexual behaviour among youth in sub-Saharan Africa.

### Strength and limitations of this study

The strength of this study is that it uses a nationally representative sample of never-married young people. Furthermore, the use of a multilevel analytical approach demonstrates the contribution of the neighbourhood and individual-level factors. And investigating variations in youth engagement in risky sexual behaviour was attributed to the neighbourhood and individual-level factors. However, because this is a cross-sectional study, we cannot determine causation. Nonetheless, our findings demonstrate the neighbourhood and individual-level factors associated with risky sexual behaviour in South Africa and the variations in risks of sexual behaviour involvement associated with neighbourhood differences. As a result, these findings are critical for developing communities-specific interventions that may lead to awareness of the dangers of high-risk sexual practices. The study is based on a quantitative survey, so it overlooks the qualitative neighbourhood and individual aspects of risky sexual behaviour. The study suggests that future studies will look at these aspects.

## Conclusion

This study revealed that about 56% of females and 61.7% of male youth had multiple sexual partners, while 39.4% of females and 23.2% of males had unprotected sex (not using a condom). Therefore, both neighbourhood and individual-level characteristics were important in explaining the involvement of young people in risky sexual behaviour in South Africa. We find that 4–10% of the variability in sexual risk-taking may be attributed to differences between communities, while 24–86% of the variations in risky sexual activities across communities is due to neighbourhoods and individual-level factors. Meanwhile, the neighbourhood and individual predisposing factors in sexual risk behaviours were: being older than 20 years, educated youth, employed/working, residing in a household with five members and above. Others were: ethnic diversity, male/female youth, living in a female-headed home, residential instability, and media exposure to contraceptives were all associated with risky sexual behaviour among never-married youth in South Africa. This study recommended that sexual risk reduction programs ought to be advanced considering the specific cultural environment due to the residential location or ethnic/race affiliation. These can be achieved by using strategies that encourage communities to challenge the social disorganisation factors that may expose young people to risky sexual behaviour. The engagement of stakeholders at the community levels is necessary for achieving this recommendation. Therefore, it presumes a wide disparity between the neighbourhood and individual-level factors and risky sexual behaviour among young people that need to be bridged. There is a need to review policies regarding sexual and reproductive health and sexuality education at the community levels among youth in South Africa.

## Data Availability

South Africa Demographic and Health Survey (2016) datasets are freely available at https://www.dhsprogram.com/data/dataset.

## Appendix 1 Variable name and classifications

**Table:**

S/No	Types of variable	Name of variable	Questions	Code
1	Dependent	Multiple sexual partnerships	Youths were considered asked if they had multiple sexual partners in the 12 months preceding the survey	Coded "1" if the response to this question was one or more partners, and if otherwise, “0”
2	Dependent	Unprotected sex (non-condom)	Did you use a condom during last sex with most recent partner?	Unprotected sex variable was coded “1” if youths reported not to use condom, and if otherwise, “0”
3	Independent	Age	Youth were asked the aged category they belong	Age was categorized as 15–17, 18–19 and 20–24 years
4	Independent	Education attainment	Youths were asked if they were employment	The educational attainment was classified as 'primary or less education, "secondary education,' or 'higher education
5	Independent	Employment status	Youths were asked if they were employment	Coded as ‘no’ or ‘yes’
6	Independent	Household size		Categorized as 1–4, 5–6 and 7 + members
7	Independent	Place of residence	Rural or urban residence	Categorized as “urban and rural”
8	Independent	Province of residence	The geographical location of the respondent	Western Cape, Eastern Cape, Northern Cape, Free State, Kwazulu-natal, North West, Gauteng, Mpumalanga and Limpopo
9	Independent	Community poverty	This was calculated using the percentage of households in the wealth index's poorest quintile	Classified as 'low' or 'high
10	Independent	Ethnic/racial diversity	This will be derived from the population group while ethnic heterogeneity will be an index of ethnicity derived from the home language spoken	Categorized as “homogenous and Heterogeneous
11	Independent	Residential mobility/instability:	This was measured by percentage of residents in a community who have moved from current place of residence five years prior to the 2016 SADHS	Categorized as “No and Yes”
12	Independent	Family structure	Sex of head of household	classified as ‘male headed’ or ‘female headed’
13	Independent	Community literacy level	The level at which a community can read and write	Categorized as “low and high”
14	Independent	Contraceptive media exposure	This was measured by whether respondents reported hearing contraceptives messages on the radio or TV or reading them in magazines in the previous few months	Coded ‘1’ if the respondent answered ‘yes’ to at least two of the media forms and ‘0’ if they did not

## Abbreviations

AIDS: Acquired immunodeficiency virus
aOR: Adjusted odds ratio
BCC: Behavioural change and communication
DHS: Demographic and health survey
HIV: Human immunodeficiency virus
MSP: Multiple sexual partnerships
NDoH: National Department of Health
OR: Unadjusted odds ratio
RC: Reference category
RSB: Risky sexual behaviour
SADHS: South African Demographic and Health Survey
SAMRC: South African Medical Research Council
SDT: Social disorganisation theory
SSA: Sub-Saharan Africa
Stats SA: Statistics South Africa
STIs: Sexually transmitted infections
VPC: Variance partition coefficient
WHO: World Health Organization
